# Production and purification of TGFb-1 in CHO-Cells

**DOI:** 10.1186/1753-6561-5-S8-P134

**Published:** 2011-12-16

**Authors:** Estabraq Abdulkerim, Sabrina Baganz, Axel Schambach, Cornelia Kasper, Thomas Scheper

**Affiliations:** 1 Institute of Technical Chemistry, Leibniz University of Hanover, 30167 Hannover, Germany; 2 Department of Experimental Hematology, Hanover Medical School, 30625 Hannover, Germany

## Introduction

The development of chemically well defined media is a demanding task in order to create the optimal conditions for an in vitro stem cell (SC) proliferation and differentiation system. Signals that govern SC differentiation into multiple mature cell types are provided by growth factors. TGFb-1 regulates a number of biological processes, including cell differentiation and proliferation, embryonic development, apoptosis and immune responses, together with cell surface receptors and signal transduction molecules within the cell. The whole signal transduction pathway leads to the specific production of distinct proteins. In this work, TGFb-1 fragment (A280 – S391) is produced using a tailor-made CHO cell line (CHO^SFS^) and purified.

## Results

**Figure 1:**
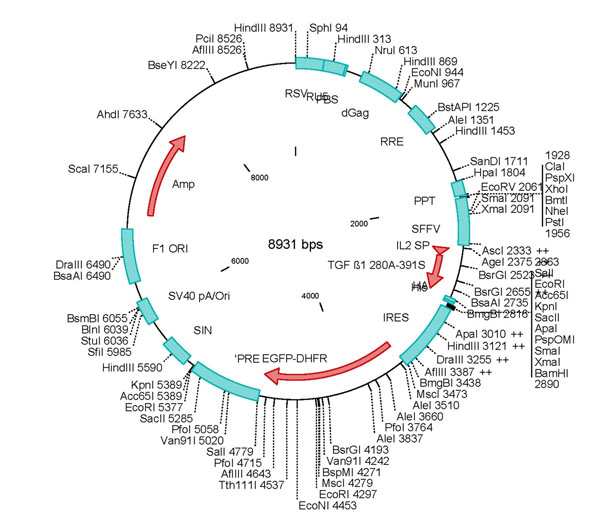
TGFb-1 vector used for the lentiviral transduction of CHO cell line (CHO^SFS^).

Chinese Hamster Ovary cells have been subjected to lentiviral transduction of TGF beta 1 vector (figure 1) resulting in the expression of His- and HA-tagged protein from (CHO^SFS^) cells. This transduction was accomplished at the department of Hematology, Hanover Medical School [1].

### Production test for TGFb-1

The cells were verified via flow cytometry for the successful transduction. The selected method involves the specific intracellular detection of His-tag by the help of fluorescence-labelled antibody.

The fixation of 2*10^5^ cells was performed by 4 % paraformaldehyde solution, cells were permeated with 0.1 % saponin followed by an incubation of the cells in 100 µL primary antibody. Afterwards the antibody was coupled to a Phycoerythrin (PE) labelled secondry antibody with a fluorescent character.

To control staining specifity, non trasfected CHO cells were used as negative control which was treated with the same procedure as transfected cells. The results of the staining show that about 77% of the cells are successfully transfected.

For the localisation of TGFb-1 cell culture supernatant and cell pellets after lysis via ultrasonic, were analysed via western blot using a combination of mouse-anti-His-tag and goat-anti-mouse-IgG-AP-conjugate. The results indicate that only a part of the protein is secreted extracellulary and the rest is present intracellulary.

### Cultivation of CHO cell line (CHO^SFS^)

CHO cells were cultured in serum free ProCHO 5 with 1% penicillin/streptomycin (PAA) and 2% L-glutamine solution (4mM). A 250 ml spinner flask (rpm. 80) was used for cell growth starting with a cell density of 0.4 * 10^6^ cells*ml^-1^ and a volume of 100 ml. The cultivation was carried out for 108 hours and medium was changed every 2-3 days. Samples were taken every 24 hour to determine cell density and viability. A maximal cell density of 1.8*10^6^ cells/ml could be achieved with a viability of 80 %.

### Purification of TGF beta 1

The supernatant of the culture was used, to perform the downstream processing.

The cells were separated by means of centrifugation and the clean supernatant was purified via heparin affinity chromatography (HiTrap TM Heparin HP columns, GE Healthcare).

The elution was performed using the binding buffer (10 mM NaH_2_PO_4_, pH 7) in addition to a linear NaCl gradient (max. 2M).

Afterwards all protein containing fractions obtained by FPLC were analysed using silver stained SDS-PAGE. The results show that TGFb-1 could be purified with a purity of 70-80 %.

## Conclusion

The production and secretion of TGFb-1 in the CHO cell line (CHO^SFS^) was successfully performed. The purification of protein was achieved using heparin affinity chromatography. Further upscaling of the procedure will be performed for achieving higher yield of the targeted protein.
